# TB or not TB! That’s the question

**DOI:** 10.11604/pamj.2019.32.126.14385

**Published:** 2019-03-15

**Authors:** Ahmed Nugud, Assmaa Nugud

**Affiliations:** 1 Aljalila Children’s Hospital, Al Jaddaf, Dubai, United Arab Emirates; 2 RAK Medical & Health Science University, Al Juwais, Al Qusaidat, Ras Al Khaimah, United Arab Emirates

**Keywords:** Infectious disease, meningitis, TB

## Image in medicine

Twenty seven years old lady presented to the emergency department accompanied by family members with complaint of fluctuating consciousness and fever for the past three days. Upon further questioning the patient has been complaining of headache for two months, decreased appetite for the past month and had a travel history to Ethiopia five months ago. Family members were not sure about any history of weight loss, the patient took a one week course of antibiotics for tooth infection two weeks ago. Physical examination showed an ill looking, vitally stable lady on chest auscultation diffuse crackles heard bilaterally. Septic work up including CSF sample was ordered to rule-out meningitis chest X-ray was also ordered. Chest X-ray result showed a clinical image specious of miliary TB (A) and results were confirmed by a positive acid fast stain of the CSF. Patient was taken to isolation and proper management was started with a final diagnosis of TB meningitis. A follow-up MRI showed few hyper-intense foci in deep white matter of left frontal lobe (B).

**Figure 1:**
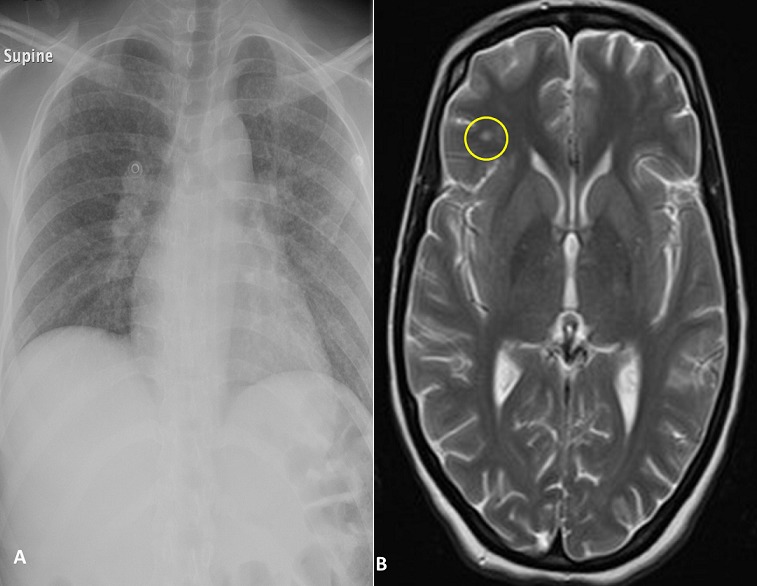
(A) chest X-ray showing bilateral hilar opacities, with suspicious miliary infiltrates; (B) brain MRI showing hyper-intense foci in left frontal lobe brain matter

